# High HOMA-IR Index in Healthcare Shift Workers

**DOI:** 10.3390/medicina55050186

**Published:** 2019-05-22

**Authors:** Caterina Ledda, Diana Cinà, Serena Matera, Nicola Mucci, Massimo Bracci, Venerando Rapisarda

**Affiliations:** 1 Occupational Medicine, Department of Clinical and Experimental Medicine, University of Catania, 95123 Catania, Italy; serena.matera@yahoo.it (S.M.); vrapisarda@unict.it (V.R.); 2 Clinical Pathology and Clinical Molecular Biology Unit, “Garibaldi Centro” Hospital of Catania, 95124 Catania, Italy; dianacinact@gmail.com; 3 Occupational Medicine, Department of Experimental and Clinical Medicine, University of Florence, 50134 Florence, Italy; nicola.mucci@unifi.it; 4 Occupational Medicine, Department of Clinical and Molecular Sciences, Polytechnic University of Marche, 60100 Ancona, Italy; m.bracci@univpm.it

**Keywords:** insulin resistance, metabolic syndrome, diabetes, shift work, night work, circadian rhythm, workers

## Abstract

*Background and objectives:* Evidence shows that shift work may be correlated with insulin resistance (IR). Therefore its estimation in clinical and prevention practice is of great significance. A cross-sectional study was performed to examine the Homeostasis Model Assessment-Insulin Resistance (HOMA-IR) Index among healthcare shift workers (HCSW). *Materials and Methods:* A total of 272 healthcare workers (HCWs) were invited to participate in the study within an occupational surveillance framework, 137 were HCSW while 135 were healthcare non-shift workers (HCNSW). Fasting glucose, insulin, and HOMA-IR Index were evaluated in each participant and correlated with shift workers. *Results:* Indicators of glucose metabolism were significantly higher in HCSW *p* < 0.001, and logistic regression analysis confirmed a significant positive association between increased values of HOMA-IR Index and shift workers (*p* < 0.05). *Conclusions:* Shift work could be a risk factor in developing insulin resistance and metabolic syndrome.

## 1. Introduction

Shift work (SW) is a work plan involving irregular or atypical hours, compared to those of a standard daytime work timetable [1]. Health implications of SW have been investigated [2,3,4,5,6,7,8,9,10], and several studies have correlated SW with chronic disease, i.e., cerebrovascular disease (CVD), coronary heart disease (CHD), diabetes, cancer, etc. [2,3,4,5,6,7,8,9,10].

Recent epidemiological studies have highlighted that SW is connected to an augmented risk of type 2 diabetes [11,12,13,14]. SW is usually related to chronic sleep defeat, which unfavorably affects glucose tolerance and cardiovascular function [15,16,17,18].

Insulin resistance (IR) is defined as a blunted response of insulin–tissue targets to insulin [19]. The spectrum of metabolic disorders associated with IR extends to pathologies other than type 2 diabetes and comprises dyslipidemia, hypercoagulability, hypertension, and inflammation, all of which are connected to the metabolic syndrome and are risk factors for cardiovascular illness [19]. Nagaya and colleagues [1] propose that SW may be related to metabolic syndrome (MS).

The estimation of MS in clinical and prevention practice is of great significance. The Homeostasis Model Assessment-Insulin Resistance (HOMA-IR) is extensively used as a method to assess MS. The HOMA-IR index computation is based on the determination of fasting glucose and insulin values [20,21].

Therefore, the present study aimed to examine the HOMA-IR Index among healthcare shift workers (HCSW) in order to contribute to evaluating the economic burden of MS in the shift workers population.

## 2. Materials and Methods

### 2.1. Study Population

This cross-sectional study was carried out from May through November 2018 under the occupational surveillance framework in two Hospital sites in the city of Catania (Italy). The research was carried out as a component of periodic occupational health surveillance. 137 HCSW and 135 healthcare non-shift workers (HCNSW) were randomly observed and invited to take part in this study. SW was defined as the work performed on three shifts, including night work, which varied during the week, 24 h, each day of the week (often abbreviated as 24/7). The study included shift workers who had done at least five years of shift work. Participation was voluntary and anonymous. The healthcare workers were recruited on their periodic occupational health checks. Exclusion criteria were the following: drugs assumption, and the presence of systemic diseases, i.e., CHD, CVD, cancer, and diabetes. The study was carried out in agreement with the procedure of the Declaration of Helsinki and the dealings were accepted by the Ethical Board of the University-Hospital of Catania (n. 737/2014 of 21-05-2014). All healthcare workers (HCWs) signed informed consent to contribute to the investigation. HCWs were questioned by a trained occupational physician and medical records, socio-demographic data, information about smoking habits, alcohol consumption, and occupational history were collected. Subsequently, a medical examination, including blood pressure, arterial measurement, and heart rate, was performed. Routine laboratory tests were added to the medical check-up.

### 2.2. Laboratory Analysis

Venous blood (5 mL) was collected from all workers in the morning following overnight fasting. The shift workers took a blood test after at least three days of detachment from the last night shift. One tube of the serum (Vacuette, Greiner Bio-One, Kremsmünster, Austria) was collected. Following this, the tubes of serum were left in a vertical pose for at least 30 min at room temperature, but for no more than 60 min. Afterward, tubes were centrifuged at 3500 rpm for 10 min, and then the serum was isolated. Fasting glucose and insulin levels were measured in order to evaluate the HOMA-IR Index. All analyses were performed with immunoassay tests (Beckman Coulter, Brea, CA, USA) on the same day of collection. The instruments were accustomed, and internal quality was performed as prescribed by the manufacturers. Reference values as proposed by the Mayo Clinic Medical Laboratories were used for all blood tests [22].

### 2.3. Statistical Analysis

To facilitate advancement in statistical analysis, HCWs were grouped in relation to their tasks: physicians, graduate sanitary (biologists, physicists, chemists, psychologists), nurses and midwives, healthcare assistance staff (physiotherapists, ophthalmologists, speech-therapists), and healthcare diagnostic staff (lab technicians, radio technicians, audiometric technicians, neuro-physiopathology technicians). Next, workers were separated by employment setting: surgery, medicine, and services. The latter comprised support departments like anesthesiology, radiology, and laboratories.

The Kolmogorov–Smirnov test checked for normality. Outcomes were described as the mean and standard deviation or as frequency and percentage. *T*-Test and Chi-square were used to evaluate the means and frequencies, respectively. The HOMA-IR Index was calculated as Matthews [20] reported.

Logistic regression and log-linear models were used to investigate the associations between the HOMA-IR Index (independent variable) and employment condition (dependent variable) and were adjusted for age, smoking habits, and alcohol consumption. Odds ratios (OR) and 95% confidence intervals (CI) were estimated. Statistical significance was set at *p* < 0.05 (two-tailed).

Statistical analysis was performed by SPSS Statistics 23.0 (IBM, Armonk, New York, NY, USA).

## 3. Results

In this investigation, 280 HCWs were examined during the occupational surveillance, 272 workers (97% response rate) agreed on taking part in the study, but 3% (*n* = 8) of the HCWs refused to give their contribution.

133 (49%) were males, 137 (about 50%) were HCSW and 135 HCNSW, their mean age was 40.1 ± 8.3 years with an age of employment of 10.9 ± 6.9. The characteristics of the HCWs are reported in Table 1.

More than 80% of the HCWs were physicians (38%) and nurses and midwives (43%). The residual part (19%) was the healthcare assistance staff, healthcare diagnostic staff, and graduate sanitary staff. The population investigated was regularly distributed in the three work settings: surgery, medicine, and services. On the medical check, no participant reported any signs and/or symptoms of illness, and routine laboratory test values fell within normal ranges for all the subjects of the two groups (data not shown).

Table 2 shows the specific characteristics of HCSW and HCNSW groups.

The two groups were homogeneous as to the variables investigated: age, gender, duration of employment, Body Mass Index (BMI), smoking behavior, and alcohol use. BMI average values fell within the normal range (18.5–24.9) in both groups.

Mean levels of fasting glucose and insulin in HCSW and HCNSW fell within the reference values (70–120 mg/dL and 2.6–24.9 µIU/mL, respectively). However, in HCSW fasting glucose and insulin levels were significantly higher (*p* < 0.001) than in HCNSW.

Mean HOMA-IR Index levels in HCSW were above the cut-off (normal range: 2.5) and significantly higher (*p* < 0.001) compared to HCNSW.

Logistic regression analysis showed a significant positive association between increased values of HOMA-IR Index and SW (*p* < 0.05) OR 1.15 (IC: 1.11 ± 1.19).

## 4. Discussion

Twenty-four hour services are an increasing part of contemporary society. Essential services (i.e., hospital assistance) are provided nonstop, and several industries and business organizations work on a 24 h basis to meet the frequently shifting demands of the contemporary world [2,23,24,25]. Therefore, companies need workers to work incessantly, creating a demand for the shift- and night-work organization. An could be expected, 20% of the powerfully active populace in the USA and the EU are occupied in various sorts of shifts involving night work [2,23,24,25]. There is substantial attention among the scientific community to studies concerning the mechanisms implicated in the health and illness of these workers, such as metabolic processes.

Shift workers, especially night workers, are inclined toward having unhealthy life habits such as smoking, poor diet, and sedentary behavior [23,26,27].

The emergent significance of shift and night work in the demands of contemporary get-it-together culture creates a burning requirement to investigate the outcomes of such schedules on workers’ wellbeing.

The effects of SW on health have only been slightly investigated, but the latest findings suggest that such schedules are likely to influence glucose tolerance and stimulate obesity and systemic arterial hypertension [28,29,30,31,32].

Clinical investigations published in the past years have addressed the associations between shift/night work and MS. These investigations comprise the association between SW and MS, diabetes, and dyslipidemia.

MS is defined as a group of IR markers, i.e., high blood pressure, impaired glucose metabolism, high serum triglyceride, and low serum High Density Lipoprotein (HDL), which result in triggering resistance to insulin-stimulated glucose uptake [33]. As MS raises the risks not simply for CHD and diabetes but moreover for CVD, several cancers, dementia, and death [34,35,36], it could be a full risk index for these chronic health concerns [1].

In our study results, it is suggested that SW may affect IR and induce MS. Comparable results were observed by Nagaya et al. [1] who demonstrated the association between MS and SW activity in workers younger than 50.

There is an apparent relationship among misalignment of circadian rhythms and MS. The causal mechanism of resistance is not entirely understood. However, a mixture of factors connected to SW are involved, for instance, changes in hormone levels and eating at times adverse to digestion [37].

The circadian management is such that melatonin production is constantly circumscribed to the night, regardless of the behavioral allocation of action and rest of the measured mammalian species (diurnal, nocturnal, or crepuscular species), that is, it is considered to be due to the chemical appearance of the night [38]. Furthermore, elevated creation is maintained throughout the dark phase of the light/dark cycle, provided there is no light in the environment, as light during the darkness (connected to the irradiance, wavelength, and duration) blocks melatonin release [38].

Melatonin has been considered as a critical factor for the synthesis, secretion, and act of insulin. The action of melatonin furthermore regulates the expression of transporter glucose type 4 or triggers phosphorylation of the insulin receptor. Consequently, a decrease in melatonin can be related to higher IR levels. This might explain the reasons why a number of investigations conclude by suggesting that melatonin supplementation occurs in shift workers [38].

Laboratory-based experimentations have revealed that individuals who sleep outside the usual period have reduced sensitivity to insulin, lacking a proportionate increase of insulin release [37]. The reported study clarified this concept, showing that melatonin, over and above insulin, is concerned in this development [38]. The risk of β-cell action was amplified approximately three-fold in shift workers who work at night and very untimely shifts [39,40].

Eating outside of the usual time of the action time, going on the feeding patterns of shift workers, may initiate a circadian misalignment, while additional investigations are needed to provide further confirmation about the function of nutrition [26,27,28,29,30,31,32,33,34,35,36,37,38,39,40,41]. This motivation pathway may activate the progress of diseases, for instance MS, regardless of factors such as job strain and physical activity [42,43]. In our study, we did not indicate eating habits, especially on night shifts, let alone the role of job strain and physical activity.

Shift workers have changed eating habits, higher energy ingestion, and amplified consumption of saturated fat and foods by a high glycaemic index [44,45]

Wefers and colleagues [46] showed that circadian misalignment, which is seen in workers who take on SW, causes a significant decrease in muscle insulin sensitivity. Previous epidemiological investigations show that night-time workers have an amplified risk of rising Type 2 diabetes mellitus [43,46,47]. Moreover, animal models with liver-specific or muscle-specific disruption of clock genes increase MS. But, circadian misalignment metabolic results in humans’ skeletal muscles are so far still unidentified [46,47]. In addition, they demonstrated on healthy participants that, owing to circadian misalignment, core clock genes expression (BMAL1, CRY1, and PER2) reserved a similar expression pattern as in the managed state [43,46,47]. This observation recommended that the molecular clock in skeletal muscles is misaligned in relation to behavioral circumstances. Additional analyses showed that genes with protein products concerned in fatty acid metabolism and PPAR signaling had amplified expression, suggesting that circadian misalignment up-regulated lipid metabolism inside muscles [46,47].

Experimental investigations have highlighted multiple molecular and cellular mechanisms through which elevated insulin levels or IR cause blood pressure to raise [48,49,50,51].

Moreover, environmental stimuli and metabolic stress correlated with changes in the chronobiological rhythms influence the neurohormonal rule of appetite and, consequently, energy equilibrium. Such instruction is mediated by central mechanisms that interrelated by the hypothalamic inhibitory stimuli of appetite such as of the adipose tissue (leptin), inferior gut (PYY3-36, oxyntomodulin, glucagon-like peptide 1-GLP-1) and higher gut (the novel peptide xenin), and helpful stimuli starting the upper gut (ghrelin) [52,53,54].

Peripheral appetite-regulating systems, as well as gut hormones, are modulated by circadian rhythm and sleep–awake homeostasis throughout sympathetic and parasympathetic nervous action and hypothalamic management of pituitary hormones [54]. SW affects sleep quality and disrupts that homeostasis, which may clarify the disrupted management of xenin and ghrelin secretion [54].

It is possible that this appetite-stimulating profile contributes to the augmented whole body adiposity and abdominal adiposity in shift workers during elevated intake of total energy, lipids, and carbohydrates. Unsurprisingly for these discoveries, night workers had lower insulin sensitivity.

In this study, the average values of blood pressures were always within normal ranges in HCSW and HCNSW.

Previous studies reported a correlation between the HOMA-IR Index and BMI [55], but in our study, the average of BMI was within normal values, without differences in either group.

It should be reported that this investigation had some limitations. Primarily, the sample size was small. Secondly, in this investigation, the consequence of changes due to exercise was not taken into account, and consequently, we were not capable of discriminating the additive effects of changes in eating performance and augmented levels of fitness on the development of glycemic control. Thirdly, we did not consider sleep disorders of HCWs, let alone the role of the job strain and physical activity.

Therefore, it will be important in the future to evaluate any possible correlation between IR and clock gene expression [56,57].

## 5. Conclusions

In conclusion, SW may be correlated with IR syndrome in HCSW younger than 50. This association may be underestimated, especially by the broad definition of SW and its effects on health workers.

## Figures and Tables

**Table 1:** Sample characteristics.

Variables	Results *n* = 272
**Gender**	
Male	133 (49%)
**HCSWs**	137 (50%)
**Age (years)**	40.1 ± 8.3
**HCWs subgroups**	
Physicians	104 (38%)
Graduate sanitary	6 (2%)
Nurses and midwives	118 (43%)
Healthcare assistance staff	23 (9%)
Healthcare diagnostic staff	21 (8%)
**Schooling**	
Bachelor’s Degree	131 (48%)
Master’s Degree	28 (10%)
Post-graduate specialization	98 (36%)
PhD	15 (6%)
**Work environment**	
Surgery	84 (31%)
Medicine	98 (36%)
Services	90 (33%)

HCSW = healthcare shift workers, HCWs = healthcare workers, PhD = Doctor of Philosophy degree.

**Table 2:** Specific characteristics of healthcare workers (HCWs).

	HCSW	HCNSW	*p* Value
Age (years)	39.8 ± 8.6	40.4 ± 7.9	n.s.*
Gender (male)	68 (51%)	65 (49%)	n.s.*
Duration of employment (years)	11.2 ± 6.8	10.6 ± 7.0	n.s.*
BMI	21.9 ± 2.1	21.4 ± 1.8	n.s.*
Smoking habits	18 (13%)	20(15%)	n.s.*
Pack/years	136 ± 20	140 ± 18	n.s.*
Alcohol consumption (g/day)	5.2 ± 4.2	6.1 ± 3.7	n.s.*
Night at work (day/month)	4.9 ± 1.3	0	<0.001
Fasting glucose (mg/dL)	94.7 ± 5.4	87.3 ± 6.2	<0.001
Insulin (µIU/mL)	12.6 ± 2.3	7.4 ± 3.5	<0.001
HOMA-IR Index	2.8 ± 0.6	1.5 ± 0.4	<0.001

* n.s. = not significant. Body Mass Index (BMI), Homeostasis Model Assessment-Insulin Resistance (HOMA-IR). Healthcare shift workers (HCSW), Healthcare non-shift workers (HCNSW).
